# The evolution of cognitive load theory and its application to medical education

**DOI:** 10.1007/s40037-015-0192-x

**Published:** 2015-05-28

**Authors:** Jimmie Leppink, Angelique van den Heuvel

**Affiliations:** 1 Department of Educational Development and Research, Maastricht University, PO Box 616, 6200 MD Maastricht, The Netherlands; 2 School of Health Professions Education, Maastricht University, Maastricht, The Netherlands

**Keywords:** Medical education, Cognitive load theory, Intrinsic cognitive load, Extraneous cognitive load

## Abstract

Cognitive Load Theory (CLT) has started to find more applications in medical education research. Unfortunately, misconceptions such as lower cognitive load always being beneficial to learning and the continued use of dated concepts and methods can result in improper applications of CLT principles in medical education design and research. This review outlines how CLT has evolved and presents a synthesis of current-day CLT principles in a holistic model for medical education design. This model distinguishes three dimensions: *task fidelity*: from literature (lowest) through simulated patients to real patients (highest); *task complexity*: the number of information elements; and *instructional support*: from worked examples (highest) through completion tasks to autonomous task performance (lowest). These three dimensions together constitute three steps to proficient learning: (I) start with high support on low-fidelity low-complexity tasks and gradually fade that support as learners become more proficient; (II) repeat I for low-fidelity but higher-complexity tasks; and (III) repeat I and II in that order at subsequent levels of fidelity. The numbers of fidelity levels and complexity levels within fidelity levels needed depend on the aims of the course, curriculum or individual learning trajectory. This paper concludes with suggestions for future research based on this model.

## Introduction

The medical domain is a complex knowledge domain. The making of a diagnosis requires medical practitioners to process and integrate information that is retrieved from multiple sources, such as the patient him or herself, fellow practitioners who have seen the patient before, the patient’s medical records, and similar patient cases. This is precisely what Cognitive Load Theory (CLT) is about: the process called learning whereby new information is digested and related to knowledge already stored and organized in long-term memory, the result of which is a more elaborate and extensive knowledge base [1].

History has taught, however, that learning does not just come about naturally. More specifically, it appears to be heavily reliant on two factors, that is, the degree of complexity of the new information to be processed, and the way in which that information is presented. To better apprehend these connections, it is important to grasp the broad workings of memory, which, throughout this review, are regarded as the ensemble of working memory and long-term memory, the former being the place where new information elements are initially received and processed, the latter being the depository where processed information is stored and organized into cognitive schemas.

CLT’s *raison d’être* will start to make sense when pointing out that working memory is limited both in capacity and duration, a finding that has found resonance in various empirical studies: only a few new information elements can be processed at a time, which elements, moreover, can—under realistic circumstances—be held in working memory for less than 20 s [2, 3]. The theory’s key remit, then, is to explain our memory processes in such a way that the available resources can be wielded as effectively as possible. Central stage in this framework are concepts such as *total working memory load*, which is tantamount to *mental effort* or *cognitive load* [4, 5] and is determined by the number of information elements that need to be processed simultaneously [1]. It goes without saying that the more working memory capacity is required for dealing with the way in which information is presented—a concept coined *extraneous cognitive load* [6]—the less working memory capacity remains for dealing with the intrinsic content of information—hereinafter referred to as *intrinsic cognitive load* [7, 8]—and vice versa.

CLT has started to find more applications in medical education research. Unfortunately, misconceptions such as lower cognitive load always being beneficial to learning and the continued use of dated concepts and methods may result in improper applications of CLT principles in medical education design and research. This review outlines how CLT has evolved and presents a synthesis of current-day CLT principles in a holistic model for medical education design. This model distinguishes three dimensions, which together constitute three steps to proficient learning. After outlining these dimensions and steps, this paper concludes with suggestions for future research based on this model.

## How CLT has evolved

As was pointed out in the foregoing, CLT revolves around the notion of a working memory that is limited in capacity and duration [1–3]. With cognitive load understood to be the sum of intrinsic and extraneous cognitive load, which to prevent cognitive overload should not exceed the narrow limits of working memory, researchers have been eager to get to grips with its essence and to find ways to measure it. The following sections will spotlight these endeavours over the course of the past decades.

### Empirical evidence for CLT principles

Empirical findings supporting CLT principles come from four types of measures: (1) indirect measures of cognitive load through task performance accuracy [9–11] or time needed for task performance [12, 13]; (2) dual-task performance measures [14, 15]; (3) bio-measures such as functional magnetic resonance imaging (fMRI) [16] or specific electroencephalographic (EEG) [17, 18] or eye-tracking variables [19]; and (4) subjective rating scales [4, 20, 21]. For a more detailed overview of findings of each of these types of measures, the reader is referred to Leppink, Van Gog, Paas, and Sweller [22]; the current review briefly highlights findings that have had a profound impact on CLT’s evolution.

Indirect measures have provided support for the previous assertion that less working memory capacity remains if more capacity is taken up by either extraneous or intrinsic cognitive load. Firstly, one found that students tend to demonstrate more accurate task performance [9, 10] and need less time for task performance [12, 13] if a strategy employed demands less problem-solving search. Especially among novice learners, engaging in problem-solving search contributes to extraneous cognitive load, leaving less room for dealing with intrinsic cognitive load, resulting, in turn, in less accurate task performance or an increase in time needed for task performance. Conversely, another study found that increased error rates in task performance could point to an elevated intrinsic complexity of information [11]. Excessive administration of intrinsic cognitive load, even with extraneous cognitive load being kept to a minimum, resulted in a very high overall cognitive load—and perhaps cognitive overload—with an increase in error rates as a logical consequence.

Further evidence in favour of a limited working memory resources model comes from dual-task studies, in which participants are instructed to simultaneously perform a primary task and a secondary task that is typically unrelated to the primary task. Learners who need to allocate fewer working memory resources to the primary task have more working memory resources available for more accurate or faster performance on the secondary task [14, 15].

Researchers using bio-measures have found, amongst others, increased activity in the dorsolateral prefrontal cortex during information processing [16], and in eye-tracking studies, negative correlations of saccade rate and amplitude with cognitive load [23, 24] and a positive correlation between fixation length and cognitive load [25].

One of the difficulties with particular bio-measures, for instance pupil dilation, is that its relation to cognitive load may vary with age [26]. Further, the approaches hitherto employed are not always practicable in the sense that they are heavily task-related, and mostly require special equipment and an even more careful study design and planning. In an educational setting this is not always feasible, which provides a logical explanation for their infrequent use so far. Subjective rating scales that measure cognitive load *experienced* by the learner are much easier to use and frequently encountered in the literature. The first scale made its appearance in the early 1990s in the form of a 9-point one-dimensional mental effort rating scale [4]. A more inclusive variation of this scale may be represented by the NASA task load index (TLX) [27], which seeks to capture five dimensions: mental, physical and temporal demands, own performance, and effort and frustration.

Apart from practical challenges, some conceptual challenges appeared. The two-factor framework one had relied on so far, that is, the division of cognitive load into intrinsic and extraneous cognitive load, did not appear to hold when considering that in some cases an increase in cognitive load could bolster learning. It therefore appeared plausible that a third type of cognitive load was involved, which in some sort of way was beneficial to learning. This concept came to be known as *germane cognitive load* [28]. Incorporating germane cognitive load into the framework, however, did not solve the riddle. On the contrary, it incited the desire to find a way to measure each type of cognitive load separately. So far, the existence of distinct types of cognitive load had been largely theoretical; although attempts to measure cognitive load had been plentiful, none of these had sought to measure each cognitive load separately.

### Attempts to measure the distinct types of cognitive load

At this point, CLT rested on the assumptions that (1) extraneous cognitive load should be kept to a minimum; and (2) germane cognitive load could arise only if intrinsic cognitive load had reached a specific level [28]. In view of these beliefs, the question arose as to how a third type of cognitive load could be assimilated into a limited working memory, and more importantly, how each of the specified cognitive loads could be quantified.

This unresolved issue sparked several efforts to devise an instrument with which the various types of cognitive load could be measured [29–33]. Unfortunately, in these studies, single items instead of multiple items were used for one or more types of cognitive load. The use of multiple indicators of the separate types of cognitive load might yield a more precise measurement and might enable researchers to separate types of cognitive load more clearly than the use of a single indicator for each. Besides, none of the studies could address the so-called *expertise reversal effect* [34, 35]. Succinctly put, instructional support in a learning task that is beneficial for novice learners loses its effectiveness or even becomes detrimental as learners become more proficient in that type of task.

Unfortunately, these conceptual and methodological issues were left largely unrecognized and, instead, a return to former principles was deemed imminent [36–38]. Germane cognitive load was reconceptualised as a subtype of intrinsic cognitive load, resulting in a plea for a move back to a two-factor intrinsic/extraneous cognitive load framework. To prevent any return to former principles from being rooted in methodological flaws in previous studies, one final attempt was made to develop a psychometric instrument that might distinguish between the three types of cognitive load [20] as defined in the late 1990s [28]. A set of four coherent studies [20] appeared to provide evidence in favour of the three-factor framework. Though being the first time since the inception of CLT that such an instrument measuring different types of cognitive load received empirical support, two follow-up studies [21] failed to provide further evidence for the germane cognitive load factor. It was therefore suggested that the three factors in the instrument be interpreted to represent intrinsic cognitive load, extraneous cognitive load, and a subjective judgment of learning [21, 22]. Table 1 presents the eight items of the questionnaire that reflect intrinsic cognitive load (items 1–4) and extraneous cognitive load (items 5–8) respectively; the questionnaire can also be used without items 4 and 8 [20–22].

**Table 1:** A new psychometric instrument for the measurement of intrinsic cognitive load (i.e., items 1–4) and extraneous cognitive load (i.e., items 5–8)

All of the following eight questions refer to the activity that just finished. Please take your time to read each of the questions carefully and respond to each of the questions on the presented scale from 0 to 10, in which ‘0’ indicates not at all the case and ‘10’ indicates completely the case:
0 1 2 3 4 5 6 7 8 9 10
[1] The content of this activity was very complex
[2] The problem/s covered in this activity was/were very complex
[3] In this activity, very complex terms were mentioned
[4] I invested a very high mental effort in the complexity of this activity
[5] The explanations and instructions in this activity were very unclear
[6] The explanations and instructions in this activity were full of unclear language
[7] The explanations and instructions in this activity were, in terms of learning, very ineffective
[8] I invested a very high mental effort in unclear and ineffective explanations and instructions in this activity

### A case for a two-factor intrinsic/extraneous cognitive load framework

To rephrase the gist of CLT, learning in fact revolves around dealing with intrinsic cognitive load [22, 39]. Given the number of information elements in a task, a more proficient learner will experience a lower intrinsic cognitive load than a novice learner, because some of the information elements in the task are already part of the cognitive schema of the more proficient learner, leaving fewer new elements needing to be processed. Providing learners with a task that comprises more information elements that are not yet part of their schemas will impose a higher intrinsic cognitive load on their minds.

The contention that scaling up intrinsic cognitive load can bolster learning found support in a recent randomized controlled experiment using mixed methods [40]. In this study, Lafleur and colleagues contrasted a typical Objective Structured Clinical Examination (OSCE) with a so-called Hypothesis-Driven Physical Exam (HDPE [41]). Whereas the OSCE concerned a *part-task*examination, the latter required students to perform a *whole-task* physical examination. In this latter group, students first made a list of anticipated findings related to several competing diagnoses; they then selected and performed physical examination manoeuvres to elicit findings, interpreted these, corrected initial manoeuvres, to finally arrive at a motivated diagnosis. Throughout the experiment, extraneous cognitive load had been kept at a minimum in both groups. Intrinsic cognitive load, however, resulted somewhat higher in the whole-task HDPE condition. Curiously, this group also revealed a better performance with regard to backward and forward diagnostic reasoning, indicating that a higher administration of intrinsic cognitive load could actually be beneficial to learning.

Of course, this reasoning does not hold till cognitive overload and beyond. As we have seen before, excessive administration of intrinsic cognitive load can do more harm than good. More precisely, when the sum of intrinsic and extraneous cognitive load exhausts working memory capacity, it cannot reasonably be expected that any integration of new information elements into existing knowledge will occur. The same holds true for trivial administration of intrinsic cognitive load: when tasks are made very easy for learners in the light of the knowledge they already possess, boredom may prevail over learning [42]. By extension, we have seen that the assimilation of an additional germane cognitive load into a limited working memory was not so easy to envisage. A two-factor framework that meets all the conditions previously addressed seems much more plausible, especially so if one considers that the concept of germane cognitive load has never really found support in empirical research. The two-factor framework is therefore a logical starting point from which to proceed. How insights yielded so far can inform medical education design and research is the topic of the remainder of this paper.

## How CLT can inform medical education design

The most enlightening insight of CLT that is of relevance to the practice of designing medical education is that, to a certain extent, teachers and learners can favourably control the learning process if three conditions are observed. Firstly, extraneous cognitive load should be kept to a minimum. Secondly, the sum of intrinsic and extraneous cognitive load should not exhaust working memory capacity. Thirdly, medical education should be designed such that it stimulates learners to allocate their available working memory resources to dealing with intrinsic cognitive load. The next paragraphs provide some hands-on advice on how to accommodate these conditions into medical education design.

### Six strategies for reducing extraneous cognitive load

One of the challenges medical education designers are faced with, is how to keep extraneous cognitive to a minimum. To achieve this, six strategies have been crystallized out which are particularly geared towards neophyte learners [28, 43] and which are briefly highlighted in the following.

1. *Use worked examples*.
  Providing learners with a detailed example of a problem that has been solved with the instruction to study the example carefully reduces the problem-solving search learners would have to engage in when attempting to solve that problem autonomously. This can also take the form of a group activity, so that learners can complement each other, maximizing their knowledge base in a less demanding fashion [44].
2. *Use completion tasks*.
  The transition from worked examples to autonomous problem solving can be facilitated through the use of either partially worked examples or completion tasks that require the learner to study the solution steps worked out and independently complete the remaining steps [45]. Such a task could also be effected during surgery, having medical students observe a surgical operation and perform only a specific part of it [43].
3. *Start with non-specific goals*.
  Having to find a definite solution to a problem requires novice learners to engage in a problem-solving search that can be reduced by first confronting learners with learning tasks that have a non-specific goal. For instance, by asking learners to ‘find as many aetiological explanations for these symptoms as you can’ instead of asking them to ‘find the most probable aetiological explanations for these symptoms’ [43], they are encouraged to extend their knowledge base without being encumbered with the more strenuous task of finding a definite solution.
4. *Avoid split attention*.
  Having to divide attention between multiple sources that are divided in either space or time forces learners to attempt to process information while holding information in their working memory. For instance, with the advent of online learning environments, we should avoid spatial split attention from learners having to scroll back and forth between parts of a webpage, attempting to process new information while holding information from another part of the page that is needed to understand or solve a problem. Likewise, we should avoid temporal split attention by providing medical students with instructions on how to use a piece of equipment right when they need it instead of sometime before that [46].
5. *Respect modality boundaries.*
  Information that could best be presented visually should not be transmitted verbally. Moreover, in some situations, learners may be served optimally by dual-mode auditory/visual presentations rather than visual-only presentations [47, 48]. For example, learners who are first confronted with specific anatomic structures may benefit from having a verbal explanation along with visual images of these structures, while verbal-only explanations may not have any beneficial effect.
6. *Avoid redundancy.*
  If a single source of information suffices to get the message across, any further information may be redundant. For instance, a diagram of the flow of blood through the heart, lungs, and body may speak for itself; if, however, it is accompanied by verbal descriptions, this presentation mode may just bring about what has been coined a *redundancy effect* [43].

With exception of the strategy aimed at avoiding redundancy, these strategies tend to be effective only for novice learners. The aforementioned expertise reversal effect [34, 35] indicates that instructional support through worked examples, completion tasks, non-specific goals, dual-mode auditory/visual presentation or avoidance of split attention may be redundant for more proficient learners and as such increase rather than decrease extraneous cognitive load. Thus, teachers and curriculum designers should monitor their learners’ proficiency in each phase of a curriculum and in a given course within a curriculum, and use this information to attune medical education design accordingly. This is necessary not only to keep extraneous cognitive load to a minimum, but also to raise intrinsic cognitive load to the most favourable level: the more the extraneous burden is eased, the more scope remains for intrinsic cognitive load to be processed. The following paragraphs outline how such desirable levels of intrinsic cognitive load can be attained.

### Three strategies for optimizing intrinsic cognitive load

Whether a learning task is regarded as complex depends on the individual learner’s cognitive schema of this type of task. The less knowledge learners can fall back on, the more complex a task will be, and, consequently the greater the amount of intrinsic cognitive load they will experience. The following three strategies can help to optimize the intrinsic cognitive load and stimulate learners to allocate their working memory resources to deal with that intrinsic cognitive load.

1. *Gradually increase task complexity*.
  As learners progress and their cognitive schemas become more developed, they are increasingly able to cope with more complex information; in fact, complex tasks are then gradually perceived as less and less complex. Hence, task complexity can be gradually upgraded.
2. *Gradually increase task fidelity*.
  To call to mind the complex task of making a diagnosis, this could be practised by having learners first review textual descriptions, subsequently work with computer-simulated patients or patients role-played by peers, then move on to simulated patients played by more professional actors, to end with real patients in an internship [43].
3. *Learning and assessment are two sides of the same coin*.
  The experiment by Lafleur and colleagues [40] illustrates that more challenging assessment criteria can influence learning for the better as long as instruction and assessment respect the boundaries of our cognitive system. Moreover, a proper assessment programme may not only stimulate learners in a particular course, but also facilitate further learning, throughout a curriculum and beyond [49].

### A model for the proficient, the neophyte, and the hapless

**Fig. 1:**
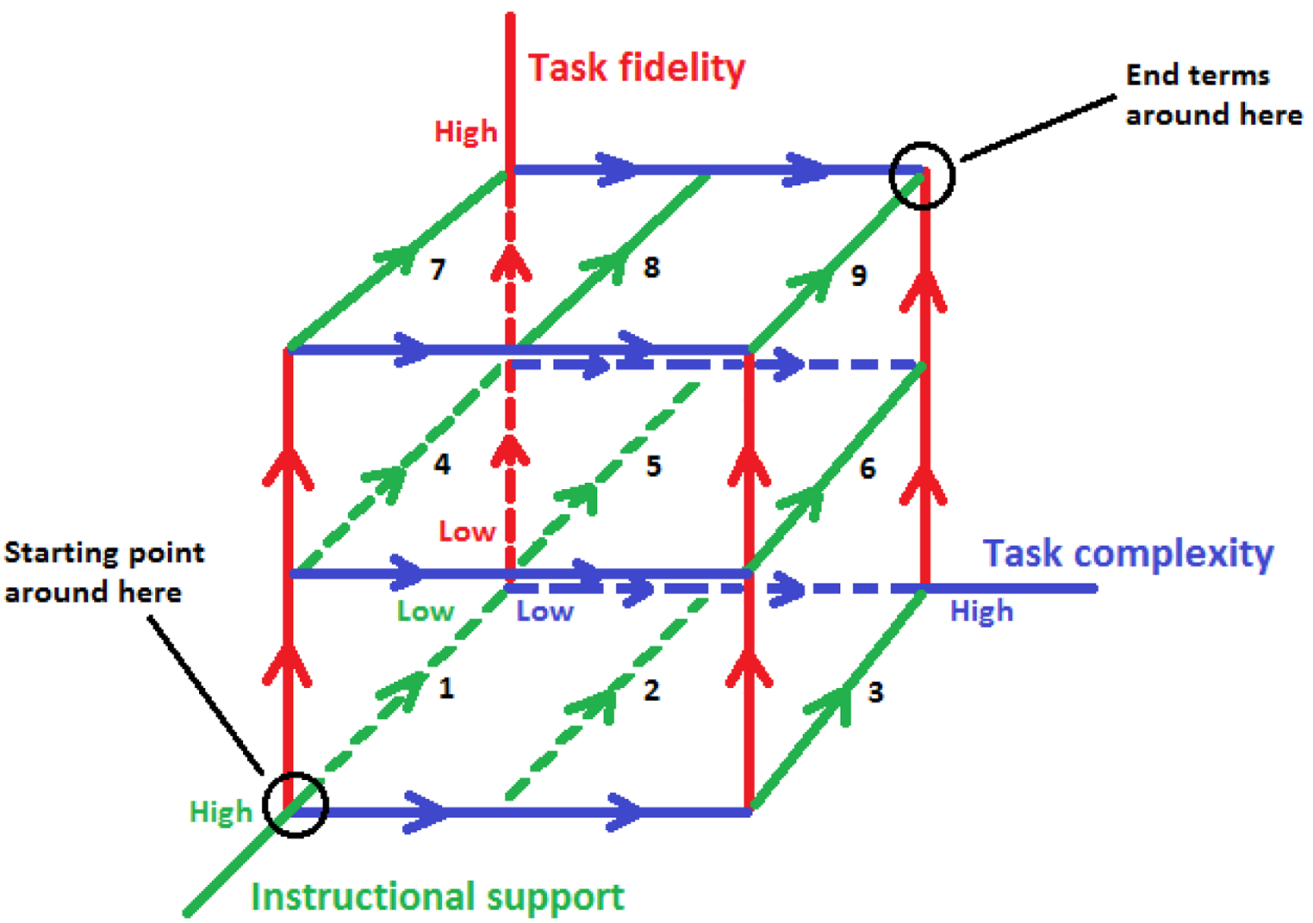
A holistic model for the design of medical education. The numbers in Fig. 1 represent the order of *green* (decreasing support) paths to walk. Thus: (1) decrease support for low-complexity low-fidelity tasks (path *1*, *down left*); (2) repeat that process for medium-complexity low-fidelity tasks (path *2*) and subsequently high-complexity low-fidelity tasks (path *3*); and (3) repeat the first two in that order (first decrease support, then increase complexity) for medium-fidelity tasks (paths *4–6*) and ultimately for high-fidelity tasks (paths *7–9*)

The aforementioned strategies come together in one coherent approach to medical education design: given end goals and assessment criteria of a course, a curriculum, one of the lines within a curriculum (e.g., skills training, research) or an individual learning trajectory, task complexity and fidelity should increase while instructional support should decrease as learners become more proficient. This approach can be visualized in a cube as in Fig. 1.

This model distinguishes three dimensions: *task fidelity* (all the way from literature to real patients), *task complexity* (the number of information elements in a task), and *instructional support* (all the way from worked examples to autonomous problem solving). These three dimensions together constitute three steps to proficient learning: (I) start with high support on low-fidelity low-complexity tasks and gradually fade that support as learners become more proficient; (II) repeat I for low-fidelity but higher-complexity tasks; and (III) repeat I and II in that order at subsequent levels of fidelity. This order of steps is based on the assumption that one needs a sufficient proficiency at lower-fidelity levels to avoid overload and disorientation at higher-fidelity levels, and sufficient proficiency at lower-level fidelity levels can be reached through an iterative process of gradually decreased instructional support and gradually increased learning task complexity.

For instance, if one cannot yet get beyond low-complexity textual descriptions of diagnoses, one is unlikely to be successful in dealing with simulated patients let alone real patients. It is like with learning a language. If one is still struggling with simple word completion tasks in a textbook, one cannot expect a more solid understanding of sentence patterns (low-fidelity task) that is needed to have a somewhat meaningful communication in that language with a teacher in class (medium-fidelity task) let alone for some meaningful communication in an everyday environment of native speakers (high-fidelity task). Likewise, if one does not understand what a standard deviation is, one cannot understand statistical tools including that concept (low-fidelity task), and consequently, one cannot expect to be able to appropriately apply these tools to simulated cases (medium-fidelity task) let alone to empirical research data or to real patient cases (high-fidelity task). One appears to need a sufficient proficiency at lower layers to continue the journey at a subsequent layer.

How many fidelity levels and complexity levels within fidelity levels are needed depends on the aims of the course, curriculum, line within a curriculum, or individual learning trajectory, and of course, the same holds for defining the relatively high-support low-complexity low-fidelity starting point; the numbers in Fig. 1 are included only to explain the basic rationale behind this holistic model. However, as the final section of this paper argues, the three steps of this model generate hypotheses that could be addressed in future research.

## Suggestions for future research

Given the implications of the distinction between intrinsic and extraneous cognitive load for medical education design, we need an increased use of intrinsic and extraneous cognitive load measures in medical education research. The instrument in Table 1 provides researchers and education designers with such measures, and these should be used along with performance indicators. Preferably, these variables should be measured repeatedly. It has been demonstrated that a one-time measurement of cognitive load using single items at the end of a series of tasks yields higher ratings than the average of multiple repeated measurements [50, 51]. Add to this the notion that cognitive load can vary substantially throughout and between learning activities [5, 20, 40], and one realizes the importance of administering cognitive load and performance measures repeatedly. Finally, one should not aggregate these measurements to one average score but treat them as they are in multilevel analysis [52] or path analysis [53] for accurate outcomes and interpretations. In the case that a larger series of repeated measurements is considered, for instance eight tasks, one could seek a combination of administering single-item ratings—such as the mental effort rating scale [4] or single items from the NASA TLX—after each task, and a multiple-item rating scale after each block of four tasks [22].

In any case, the repeated administration of cognitive load measures can help to study the guidelines provided in the previous section, which are based on more than two decades of CLT and research inspired by that theory. Further, it can help to facilitate research that seeks to expand the CLT framework for medical education purposes.

### Expanding the framework: three factors to be considered

CLT could be extended further if three factors were taken into account, the first factor being emotion. More specifically, the question as to how emotions can influence our ability to process information in a variety of circumstances is one worthy of answering. To date, this factor has received minor thought. Recent findings such as that emotion could inhibit learning and decision-making if it stimulates bias [54] or when pondering over its cause [55, 56] should serve as a point of departure for more in-depth research, for instance how emotion affects learning and task performance across levels of task fidelity as envisioned in the model in Fig. 1.

Another factor the impact of which has been equally under-researched is assessment. While CLT’s emphasis has been essentially on learning and instruction, it has been less concerned with addressing how end terms and assessment criteria can affect the extent to which we actually engage in learning. The experiment by Lafleur and colleagues [40] demonstrates that slightly more challenging end terms and assessment criteria can stimulate learners to invest more effort in learning. In the context of the model in Fig. 1, more challenging end terms and assessment criteria may result in a larger cube that comprises more levels of complexity and/or fidelity. This may undermine learning when the combination of intrinsic and extraneous cognitive load is taken to the limits of working memory but may stimulate learning when extraneous cognitive load is kept to a minimum and intrinsic cognitive load is around an optimum. The aforementioned experiment should be replicated in a variety of settings, with different kinds of courses, at different stages of a curriculum, and in different contexts of learning at the workplace to further assess this statement.

Finally, the distinction between intrinsic and extraneous cognitive load—which is crucial for medical education design—cannot be made by a single-item measure, and it is unclear to what extent the NASA TLX could help us to make that distinction. However, the NASA TLX could provide inspiration to researchers when considering for instance to build forth on the instrument in Table 1, especially in the context of learning procedures and skills. Although the instrument in Table 1 already appears to take into account mental demands, performance, and effort, the other aspects addressed by the NASA TLX are not really visible here. Including the NASA TLX in the further development of measures for intrinsic and extraneous cognitive load could result in an instrument that still enables us to distinguish between intrinsic and extraneous cognitive load but also takes into account physical and temporal demands as well as frustration. In any case, the development of new measures should involve not only *quantitative* but also *qualitative* methods. A strength of research inspired by CLT carried out since its introduction is a certain rigour in study design and procedure. However, not that many studies have included qualitative methods, and this is perhaps one of the reasons why most attempts to develop measures for intrinsic and extraneous cognitive load have failed. One of the strengths of medical education research is that one can easily find examples of excellent use of quantitative as well as of qualitative methods. We will need both in the further development and refinement of measures, even if the combined use of subjective measures along with indirect measures, secondary tasks, and/or bio-measures is considered. In fact, when feasible, studies involving different types of measures would be recommended. This could shed light for instance on how specific bio-measures relate to intrinsic and/or extraneous cognitive load.

## To conclude

This review has provided a model for medical education design along with guidelines for future research. Given the excellent context medical education delivers for extending CLT further, by incorporating factors hardly considered thus far, both medical education *and* CLT could thrive on an increased application of CLT principles.

## Essentials

- Given that the sum of intrinsic and extraneous cognitive load should respect the limits of working memory and learning is about dealing with intrinsic cognitive load, extraneous cognitive load should be minimized while intrinsic cognitive load should be optimized.
- Instructional support that reduces extraneous cognitive load among novice learners may contribute to extraneous cognitive load among more proficient learners; this instructional support should therefore fade gradually as learners become more proficient.
- More proficient learners have more elaborate cognitive schemas of a type of learning task and therefore experience a lower intrinsic cognitive load than their less proficient peers when dealing with a task of that type; learning task complexity should gradually increase as learners become more proficient.
- High-fidelity tasks such as dealing with real patients are generally more challenging than lower-fidelity tasks such as computer-simulated patients or textual descriptions of diagnoses; learning task fidelity should gradually increase as learners become more proficient.
- Measures of intrinsic and extraneous cognitive load should be used in medical education research along with performance indicators to facilitate further study and the progress of individual learners in a course, curriculum or individual learning trajectory.

### Source(s) of support in the form of grants

None
